# Species-Specific Responses to Community Density in an Unproductive Perennial Plant Community

**DOI:** 10.1371/journal.pone.0102430

**Published:** 2014-07-22

**Authors:** Michael A. Treberg, Roy Turkington

**Affiliations:** Department of Botany, and Biodiversity Research Center, University of British Columbia, Vancouver, BC, Canada; Institut Pasteur, France

## Abstract

Most studies of density dependent regulation in plants consider a single target species, but regulation may also occur at the level of the entire community. Knowing whether a community is at carrying capacity is essential for understanding its behaviour because low density plant communities may behave quite differently than their high density counterparts. Also, because the intensity of density dependence may differ considerably between species and physical environments, generalizations about its effects on community structure requires comparisons under a range of conditions. We tested if: (1) density dependent regulation occurs at the level of an entire plant community as well as within individual species; (2) the intensity (effect of increasing community density on mean plant mass) and importance (the effect of increasing density, relative to other factors, on mean plant mass) of competition increases, decreases or remains unchanged with increasing fertilization; (3) there are species-specific responses to changes in community density and productivity. In 63 1 m^2^ plots, we manipulated the abundance of the nine most common species by transplanting or removing them to create a series of Initial Community Densities above and below the average natural field density, such that the relative proportion of species was consistent for all densities. Plots were randomly assigned to one of three fertilizer levels. At the community level, negative density dependence of mean plant size was observed for each of the 4 years of the study and both the intensity and importance of competition increased each year. At the species level, most species' mean plant mass were negatively density dependent. Fertilizer had a significant effect only in the final year when it had a negative effect on mean plant mass. Our data demonstrate a yield-density response at the entire community-level using perennial plant species in a multi-year experiment.

## Introduction

Most experimental studies of density dependence in plant populations are problematic in that they focus on single species within a community and seldom consider regulation at the total community level. This raises the question of whether a community-level carrying capacity can be defined. Within multi-species plant communities, if the community is at carrying capacity then any reduction in density of one species is likely to be associated with increases in density (or biomass) of other neighboring species that are likely to be potential competitors. Knowing whether a plant community is at, or close to, carrying capacity is essential for understanding its behaviour [1] and there is reason to believe that low-density plant communities will behave quite differently, and less predictably, than plant communities close to carrying capacity [2]. It is argued that most natural plant communities are at, or near, carrying capacity, and their dynamics are therefore quite predictable because the community is using all available resources that act as a constraint on plant community dynamics [2]–[3]. These conclusions thus far do not have broad empirical support and to test them requires manipulation of whole community densities, both below and above the natural field densities, and the monitoring of individual species populations within these communities in response to altered densities [4]–[7].

Competition is important in structuring plant communities [8]–[9], but the intensity at which it occurs is dependent upon local conditions and has been the subject of much debate [9]–[13]. Much of the debate focuses on competition in low productivity communities [4] [14]–[15] and although many studies demonstrate that competition, as well as facilitation, is prevalent in a wide range of natural communities [16]–[18], they tell us little about their overall effects on the community (but see [19]) and mostly focus on the effect or response of certain species [8]. Theoretical studies suggest that individual-level data will often not predict community patterns, even on a local spatial scale [20]–[22] and empirical evidence likewise has shown that individual-level effects of competition could not predict community-level effects [23]. Rees [12] recently presented a simple framework for interpreting the results of short-term competition experiments along natural productivity gradients.

A method for directly examining the effects of competition on community structure using multi-species mixtures is the Community Density Series (CDS), first described in [24]. It is a multi-species (community) version of the traditional single-species (population) yield-density experiments [25] in which the yield of a single species is measured when the species is grown at a range of densities ranging from very low to very high. In this method, the density of an entire community is manipulated in the same way as a single species to obtain densities below and above the natural condition of the community. The lowest density plots, where density is low enough to preclude plant-plant interactions, characterize the “null” community and this can be compared to higher density plots where biotic interactions are affecting the plant community as a whole [24]. In addition, each plant species in the CDS can be considered separately to determine if they respond similarly to changing density. Another advantage of the CDS is that both negative and positive density dependent processes are detectable. The influence of resource level on these responses can also be investigated by examining changes in the yield-density relationship. For example, if limiting resources are increased, we may expect an increase in the constant final yield or an increase in the intensity of competition. An additional advantage of the CDS is that the slope and the *R^2^* from the regression of the yield-density relationship represent both the intensity and importance of competition respectively [26]. In traditional yield-density studies, the coefficient of determination, or *R^2^*, from simple linear regression [26]–[27] or it's multivariate equivalent [28]–[29] can be interpreted as being the importance of competition. *R^2^* represents the importance of competition, compared with other possible factors affecting yield, because it is the proportion of variation in yield that is directly due to the density. The ability to quantify both the intensity and importance of competition can help untangle some of the debates surrounding the role of competition in structuring plant communities [13] [16]. More recently, Bennett and Cahill [13] used a novel method (based on Lamb and Cahill [19] to estimate the intensity and importance of competition in a grassland with a limited range of productivity levels. They measured the performance of 22 different species in the field, with and without neighbours, and averaged the responses. The Bennett and Cahill [13] approach focuses on the effects of neighbours on seedling survival and growth but does not address the question of density-dependent regulation at a community level.

The CDS has been successfully applied in both experimental and natural communities of annual plants in the Negev Desert [4]–[7] [30] in an experimental bryophyte community [31] or a single season in an old-field community [23] and in an experimental boreal understory community [32]. However, it has never been applied in a perennial system in a multi-year study. Using the CDS, we investigated the influence of competition in structuring an unproductive boreal understory plant community. Specifically, we tested if density dependent regulation occurs at the level of an entire plant community i.e. if the community is at carrying capacity, or constant final yield [2] as well as among individual species; if the intensity and importance of competition changes with increasing fertilizer addition; and if there are species-specific responses to both changes in community density and community productivity. In addition, we asked at what community density does competition begin to have an effect, and, at what community density is maximum constant final yield achieved.

## Methods

### Study site

The study site is located within the boreal forest close to Kluane Lake in the southwestern Yukon Territory (138° 16′ W; 61° 00′N) at approximately 1000 m above sea level. This research was done in an area that consists of Crown Land (Scientist & Explorer permit, Heritage Branch, Yukon Government) and we have oral permission from both the Champagne and Aishihik, and the Kluane First Nations. The research did not involve endangered or protected species. This ecosystem is extensively studied for both its animal and plant components and was used for the Kluane Boreal Ecosystem Project [33]–[34]. Previous research has shown that the vegetation in this system is nutrient limited [35] and competition affects some of the understory plant species [36]. Beginning in 1995, an outbreak of spruce bark beetle caused the death of many of the overstory trees (White spruce; *Picea glauca* (Moench) Voss s.l.)) resulting in a rather open canopy. Although there are herbivores such as snowshoe hares, red squirrels and microtine rodents at this site, understory composition is more affected by the limited soil nutrients than by herbivores [34].

### Experimental design

We used a full-factorial block design with six levels of density plus control plots, 3 levels of fertilizer and three replicates per treatment, for a total of 63 plots, each 1 m×1 m. In late May 1999, the 63 plots were marked in an area of approximately 25 m×75 m. These plots were located in patches with representative samples of the vegetation common to the understory community in this forest. Plots were in small groups of 2 to 5, with a minimum of 1 m between adjacent plots. Each group of plots was surrounded by a 1 m high 2.5 cm mesh galvanized chicken wire fence.

Percent cover of all species in the 63 plots was estimated using a point quadrat frame with 100 points per plot (Table S1). The nine most abundant species that represented 94.4% of the total cover, (97.5% of biomass in control plots in 2002) were chosen to be included in the community density series (CDS). Seven of the species are herbaceous perennials: *Achillea millefolium* L. ssp. *borealis* (Bong.) Breitung (yarrow), *Epilobium angustifolium* L. s.l. (fireweed), *Festuca altaica* Trin. (northern rough fescue), *Lupinus arcticus* Wats. (arctic lupine), *Mertensia paniculata* (Ait.) G. Don var. *paniculata* (bluebells), *Senecio lugens* Richards. (black-tipped groundsel), *Solidago multiradiata* Ait. (goldenrod). The remaining two species are prostrate woody perennials: *Arctostaphylos uva-ursi* (L.) Spreng. s.l. (bearberry) and *Linnaea borealis* L. ssp. *americana* (twinflower). Hereafter we will refer to species using their generic name following the nomenclature of Cody [37].

We estimated density of six of the species in all plots by counting stems or ramets; it was not possible to estimate density for *Arctostaphylos, Linnaea* or *Festuca* so we used percentage cover instead. Using these abundance estimates we constructed a geometric series of six Initial Community Densities consisting of 1/16, 1/8, 1/4, 1/2, 1 and x2 the average natural field density. The x1 density closely approximated the density of the natural vegetation estimated from the initial survey of the community. All plots in the CDS were manipulated by transplanting and removing plants such that the relative proportion of the nine most common species was consistent for all densities. Without exception, every plot in the CDS had some plants added and some removed to obtain the proper proportions of the nine study species. To increase the density in plots, transplants were taken from the surrounding vegetation either as large sods containing many individuals (and sometimes many species) or as single shoots. Removal was accomplished by cutting the unwanted shoots off at ground level. In the lower density plots, an attempt was made to keep the remaining plants approximately equidistant from each other. Density manipulations began in mid-June 1999, and were completed by mid-July. Some regrowth of removed plants occurred but this was removed before the end of the season survey done in the final 2 weeks of August. No plants were added at this time. In 2000, minor weeding was completed in June to adjust to the desired densities. No other density manipulation was required and plants were allowed to grow for three more growing seasons.

Fifty-four of the 63 1 m^2^ plots were randomly assigned to the CDS; the remaining 9 plots were used as controls and did not have any density manipulation. All 63 plots were randomly assigned to one of the three fertilizer levels. At the beginning of each growing season, the soil surrounding all plots was cut to a depth of approximately 25 cm just outside of the 1×1 m perimeter to sever any belowground connections between plant inside and outside the plots.

Three levels of fertilization were used - the low fertilizer treatment (control) had no fertilizer added. Granular fertilizer (N-P-K; 21-7-7) was added after snowmelt, at the end of May or early June for each of the 4 years of the study. Fertilizer was added at a rate of 13 g N m^−2^ y^−1^, 4.4 g P m^−2^ y^−1^ and 4.4 g K m^−2^ y^−1^ for the medium fertilizer treatment and at double this rate for the high treatment. These are within the range of application rates from other studies in this area that demonstrated a significant effect of fertilizer addition [34]. These nutrients and the medium rate of application were used following the protocols established by the 10-yr Kluane Boreal Forest Project [33]–[34]; preliminary tests had shown N and P to be limiting and the rate of application was the lowest of the range of application rates used by foresters when fertilizing forests.

As a response variable, in each year the average plant biomass was estimated or measured. However, because we needed to estimate biomass within the plots, yet could not destructively harvest, surrogate measures of biomass were used to approximate the biomass for each species. In July 1999, 20 plots were randomly chosen nearby the experimental plots and were sampled for percent cover of each species. The plots were clipped to ground level, sorted to species, dried, and the relationship between each species cover and biomass was determined. The biomass of *Arctostaphylos*, *Festuca,* and *Linnaea* was accurately estimated by percent cover. The width of the widest leaf, length of the longest leaf, the number of leaves and maximum height were measured for random individuals of the remaining herbaceous species. These were then cut at ground level and dried. These measurements were then related to shoot mass and the best fitting relationships were determined (Table S2). All relationships were statistically significant and *R^2^* values ranged from 0.62 to 0.97. For each year of the study, during peak biomass, which occurs approximately at the end of July, all individuals in each CDS plot and control plot were measured and the plot biomass estimated (Table S3).

At the end of August 2002, all plants were counted in all plots. All aboveground biomass was removed and each individual plant was bagged separately before being air dried for transport to the University of British Columbia. All samples were oven dried at 60°C for 48 hours and weighed to the nearest mg.

### Analysis

The effects of density and fertilizer addition on the average plant mass were examined using analysis of covariance (ANCOVA) with density as the covariate and fertilizer as a categorical variable with 3 levels. All analyses were done using JMP 4 [38]. The effect of density on the understory community was analyzed using an individual performance approach [4]. By examining the average performance of the individual in relation to density, any non-zero slope would indicate density dependence. For example, an increase in performance with density, whether linear or nonlinear, would indicate positive density dependence or facilitation. A negative slope would indicate negative density dependence or competition. Because the ANCOVA requires a linear covariate, four transformations were used to linearize the data: linear, power, semilog and reciprocal. The best fitting model, with the highest *R^2^*, is reported. These transformations also assisted in making the ANCOVAs better meet the usual statistical assumptions, namely, normality of residuals, homogeneity of variances, homogeneity of regression slopes, linearity of regression, and independence of error terms. If density were not significantly related to the response variable, standard ANOVA was used to determine the effect of fertilizer addition.

At the community level, the effect of density was examined on the mean plant mass for each of the 4 years of the experiment. We used a mean plant size index calculated as the total biomass of the entire plot divided by the initial density, and the relative densities from the Initial Community Densities were used as the covariate for all ANCOVAs.

The density at which competition began to reduce the mean plant mass was determined by stepwise regression of mean plant mass against density, beginning with the low density plots. The density at which a final constant yield was reached was determined by regressing the final plot yield (total biomass) of the two highest densities against density. Lower density plots were added until the slope became significant. The last density that had a non-significant slope is the density where a final constant yield was reached.

The effect of density on the diversity in the CDS plots was also examined. The species richness of each plot did not change in the 4 years of the study; however, if density affects species differently, we may expect to see changes in their relative abundance or evenness. In the final year of the study, we used each species' mass in the plots to calculate an evenness index (*E_var_*) [39]. Evenness values close to 1 indicate that species are nearly uniformly abundant (i.e. are of similar mass) and values close to 0 indicate that one or a few species are much more abundant than the others are. Therefore, if we detect any change in evenness with changing density, there would have to be species-specific changes in abundance.

The effect of fertilizer addition on the plot biomass of the unmanipulated controls, those plots not part of the CDS, was also examined using ANOVA. The controls were also compared to the x1 CDS plots to determine if the final plot mass was similar between the manipulated plots and the unmanipulated plots and whether there was any difference in their response to fertilizer addition.

Species-specific effects of density and fertilizer addition were examined on the final mean biomass for each plant species using ANCOVA with the relative planting density being used as the covariate. If density was not related to the final mean plant mass, ANOVA was used to determine the effect of fertilizer addition.

## Results

### Community-level responses

**Figure 1:**
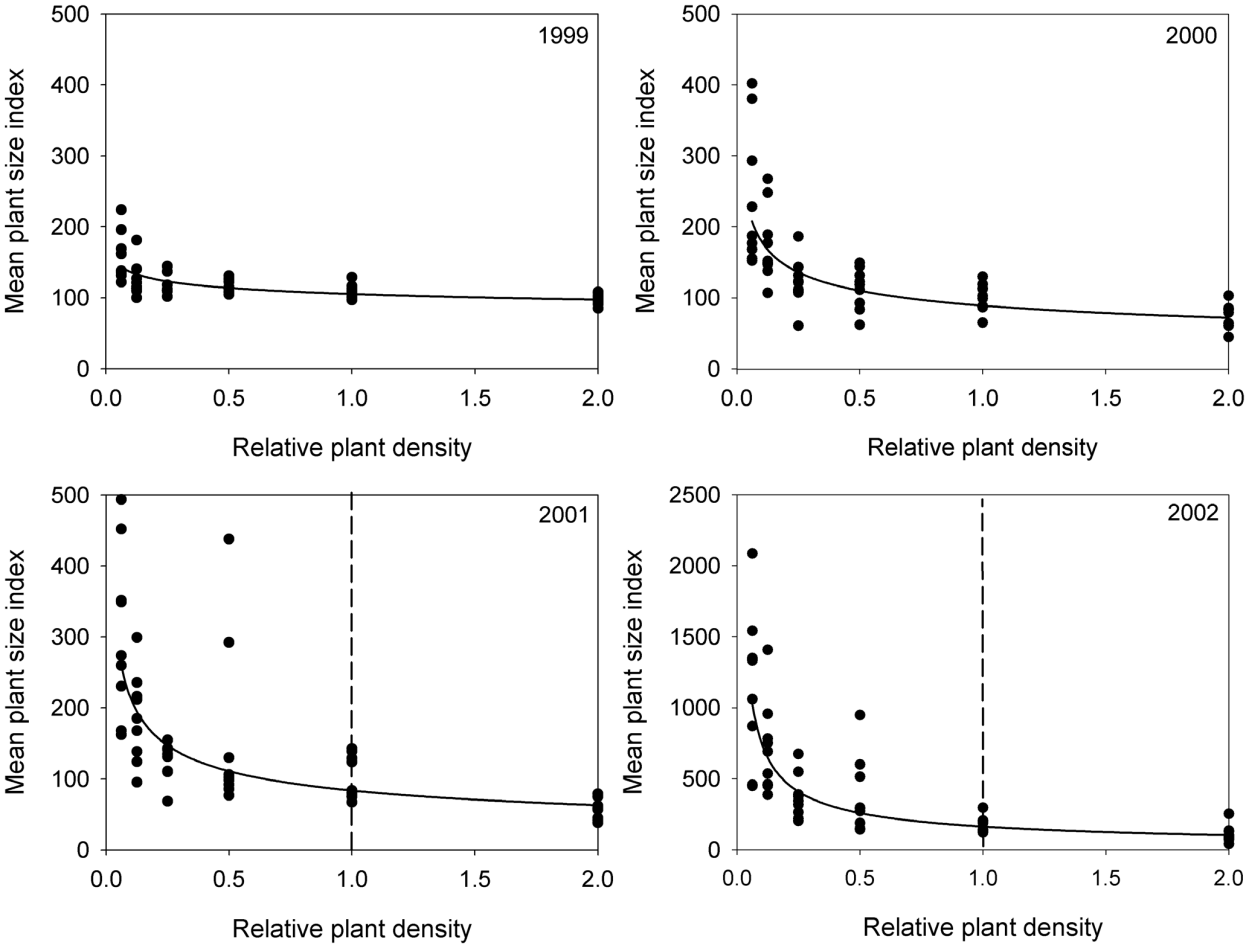
The effect of density on the mean plant size index (the total plot mass divided by density) for the years 1999 through 2002. All curves are statistically significant (P<0.001) and the coefficients for the best fit curve are given in Table 1. The y-axis for 2002 has a different scale than the graphs for other years. The natural field density is x1. The density that competition began to reduce mean plant size was at x1/8 (i.e. 0.125) for all graphs. The vertical dashed line represents the density that constant final yield is reached.

Negative density dependence of the mean plant size index was observed each year of the CDS experiment with the negative slope becoming increasingly steeper each subsequent year (Table 1, Fig. 1). Generally, mean plant size increases each year at low densities, but remains constant or decreases at higher densities. Therefore, the intensity of competition (the slope) increased each year. Similarly, the importance of competition (*R^2^*) also increased with time. In all years, the relationship between mean plant size and density was nonlinear (Table 1). Mean plant mass was significantly higher below the x1 density (the natural density of the community) (Fig. 1). The density at which competition began to reduce mean plant mass was at x1/8 for years 3 and 4. Final constant yield was only reached in 2001 and 2002 at x1, the average natural density observed in the field.

**Table 1:** Regression coefficients for the mean plant size index (total plot mass divided by the density) and density relationships for the years 1999 through 2002 and the evenness and density relationship in 2002.

Variable	Model	Intercept	Slope	*R^2^*	*P*
Mean plant size index 1999	power	4.658	−0.113	0.495	**<0.001**
Mean plant size index 2000	power	4.488	−0.306	0.617	**<0.001**
Mean plant size index 2001	power	4.427	−0.407	0.613	**<0.001**
Mean plant size index 2002	power	5.096	−0.663	0.742	**<0.001**
Evenness (*E_var_*)	semilog	0.338	5.97×10^−2^	0.309	**<0.001**

Significant values (*P*<0.05) are in **bold**. These data are plotted in Figures 1 and 3. The negative slopes indicate negative density dependence (or competition) for the plant size index. Model type refers to the data transformation that best linearizes the data. The degrees of freedom (df = 53) are for the model and error combined.

**Figure 2:**
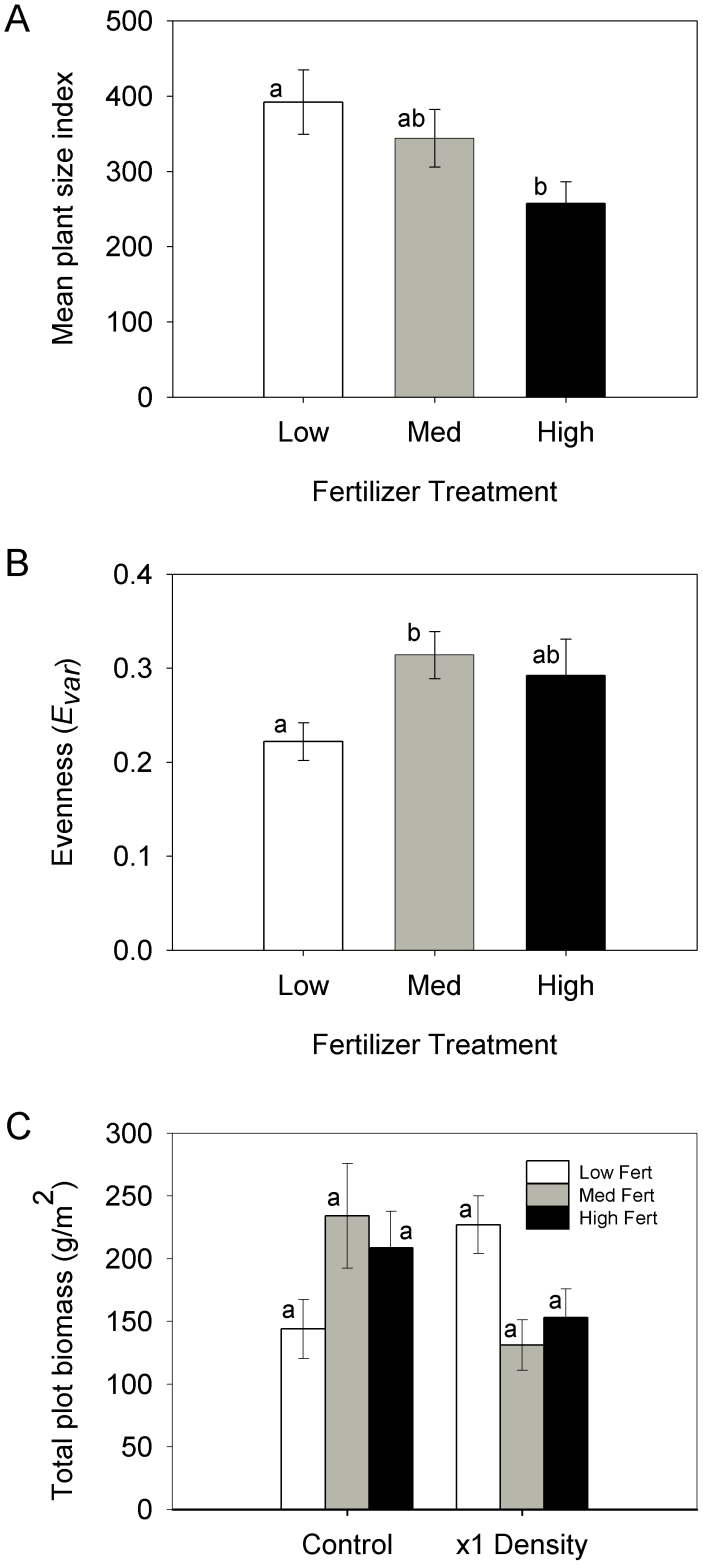
The effect of fertilizer level on (A) mean plant size index (total plot mass divided by density) in the CDS, (B) evenness in the CDS and (C) the total plot biomass for the controls (density not manipulated) and the x1 density plots in the CDS experiment. Error bars are ± 1 S.E. All data are for 2002. Columns that share the same letter are not statistically different (Tukey's HSD, *P*>0.05).

The mean plant size index was affected each year by plant density; however, fertilizer only had a significant effect in the final year (Fig. 2A), although there was a significant fertilizer and density interaction in the first year of the study (Table 2). In the final year, fertilizer surprisingly had a negative effect on mean plant size with the highest growth in the unfertilized plots (Fig. 2A).

**Table 2:** Summary of ANCOVAs for the mean plant size index in the CDS for 1999 to 2002 and ANOVA for total plot biomass in the control plots in 2002.

Variable	Source	df	SS	*F*-ratio	*P*
Mean plant size index 1999	Density	1	0.961	57.362	**<0.001**
	Fertilizer	2	0.018	0.539	0.587
	Density x Fertilizer	2	0.158	4.712	**0.014**
	Error	48	0.804		
Mean plant size index 2000	Density	1	7.077	91.225	**<0.001**
	Fertilizer	2	0.421	2.715	0.076
	Density x Fertilizer	2	0.250	1.612	0.210
	Error	48	3.723		
Mean plant size index 2001	Density	1	12.546	78.564	**<0.001**
	Fertilizer	2	0.080	0.251	0.779
	Density x Fertilizer	2	0.172	0.538	0.588
	Error	48	7.665		
Mean plant size index 2002	Density	1	33.254	162.465	**<0.001**
	Fertilizer	2	1.603	3.916	**0.027**
	Density x Fertilizer	2	0.124	0.303	0.740
	Error	48	9.825		
Evenness (*E_var_*)	Density	1	0.269	25.455	**<0.001**
	Fertilizer	2	0.084	3.977	**0.025**
	Density x Fertilizer	2	0.011	0.526	0.595
	Error	48	0.508		
Controls (Total Plot Biomass)	Control	1	3197	0.671	0.430
	Fertilizer	2	104.858	0.011	0.989
	Control x Fertilizer	2	28025	2.941	0.095
	Error	11	52406		

The control treatment for the total plot biomass ANOVA compares the mean of the unmanipulated control plots to the x1 density in the CDS plots. Significant values (P<0.05) are in **bold.**

**Figure 3:**
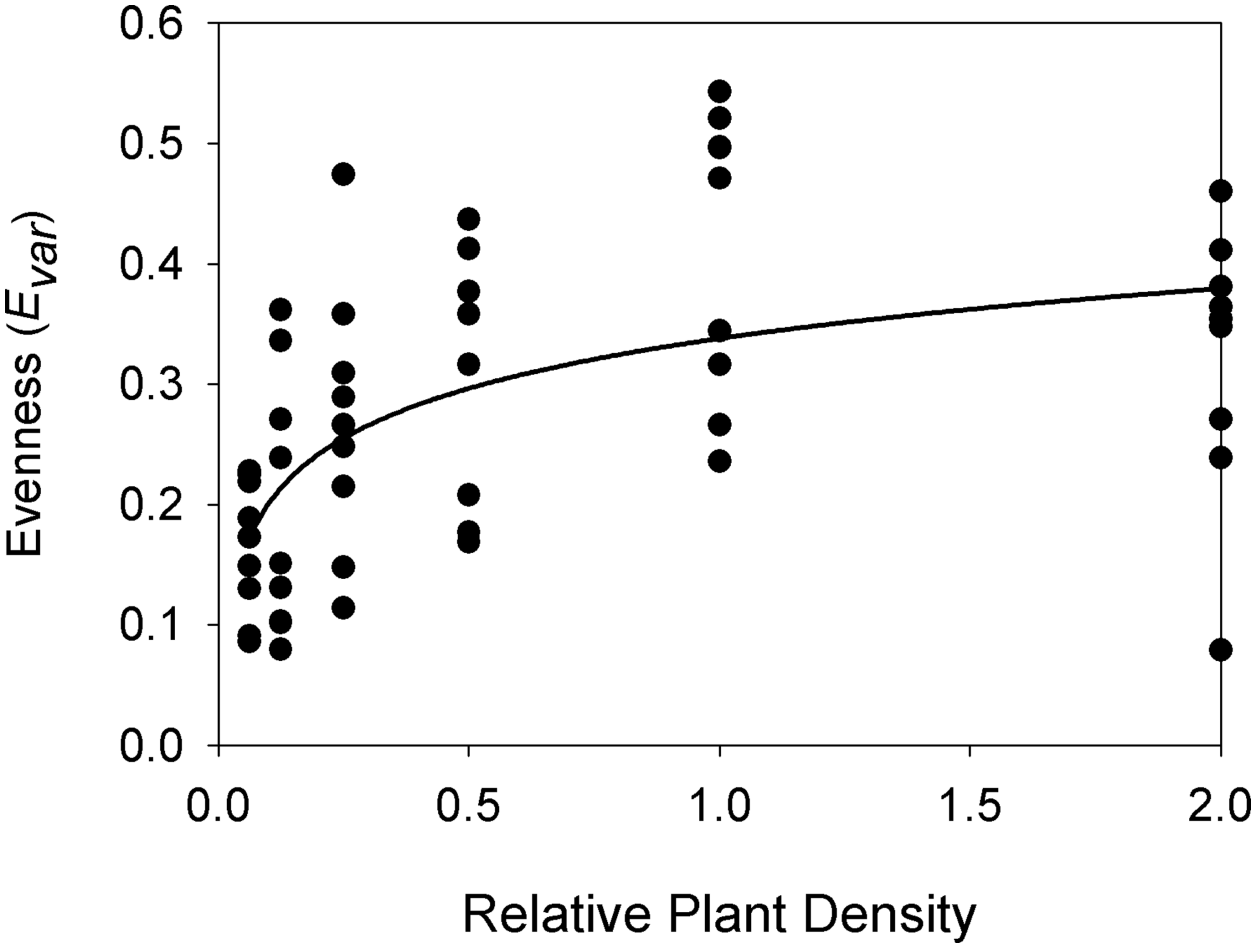
The effect of plant density on evenness (*E_var_*). The natural field density is x1. The best fit curve is statistically significant (*P*<0.001) and the coefficients for the curve are given in Table 1.

Species evenness in the community in the final year was also affected by density (Table 1, Fig. 3) with the highest evenness in the higher density plots. The significant nonlinear relationship between evenness and density means that the relative proportion of each species' biomass was not constant, i.e. the species' responses were not consistent along a density gradient. Evenness was also significantly different between fertilizer levels, regardless of density, with the lowest evenness in the unfertilized plots (Table 2, Fig. 2B).

There was no difference in the overall plot biomass between the unmanipulated controls and the x1 density plots (Table 2, Fig. 2C). There was also no effect of fertilizer addition on the plot biomass for either the control or the x1 density plots (Table 2, Fig. 2C).

### Species-level responses

**Figure 4:**
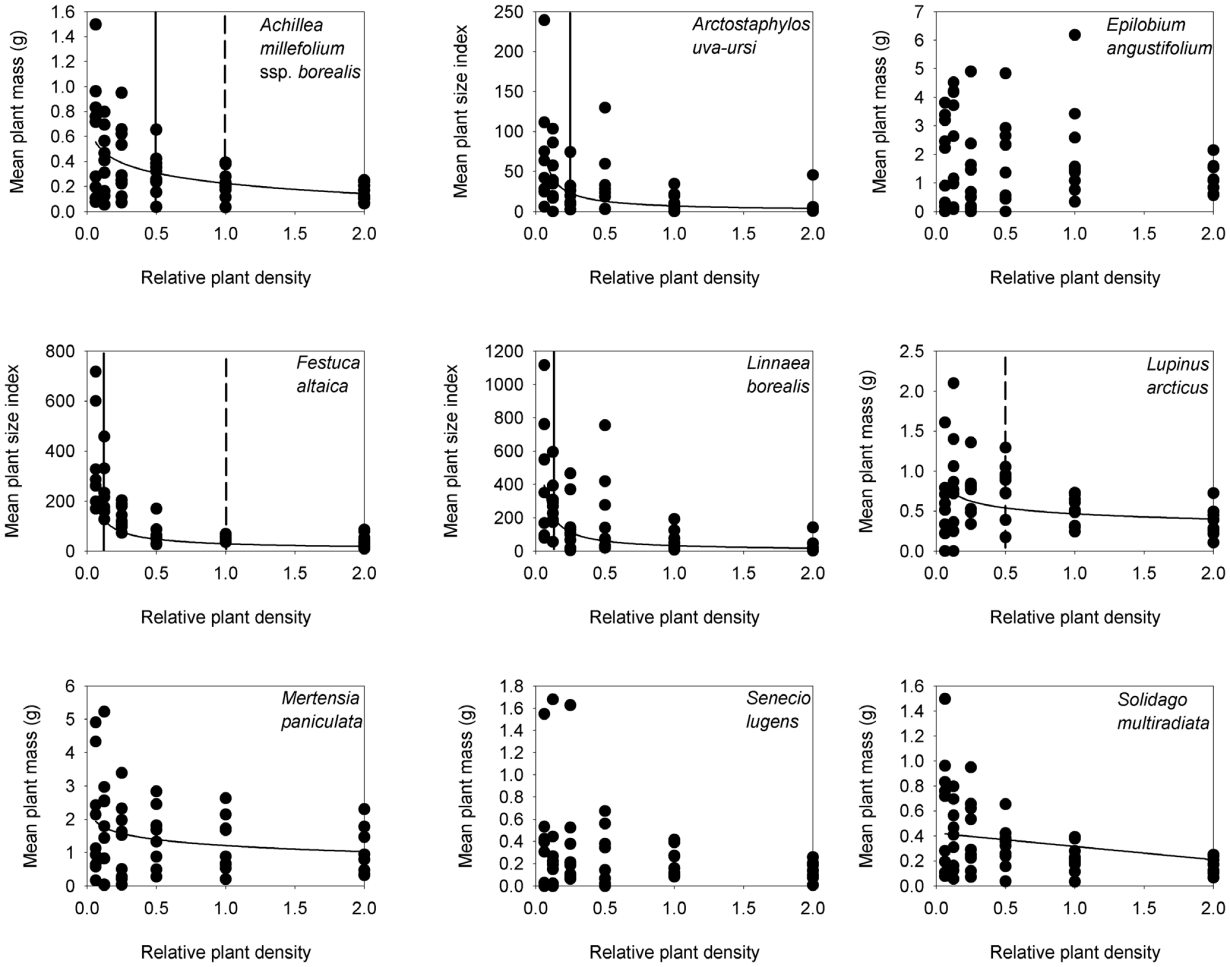
The effect of plant density on the mean plant mass or mean plant size index (total plot mass divided by density) for species in the CDS plots in 2002. All curves are statistically significant at *P*<0.05, except for *Mertensia* and *Solidago*, which are significant at *P*<0.10. The coefficients for the best fit curve are given in Table 3. The natural field density is x1. Solid vertical lines indicate the density that competition begins to reduce the mean plant mass. Dashed vertical lines indicate the density where the final constant yield for that species was reached.

Most species' mean plant mass were negatively and nonlinearly density dependent (Tables 3, 4, Fig. 4). Only two species' mean mass, *Epilobium* and *Senecio,* were not related to density, although *Mertensia* and *Solidago* were only related to density at P<0.10 (Table 3). The intensity of competition was highest on *Linnaea* and *Arctostaphylos* (slopes of −0.897 and −0.851, respectively) and was also high on *Festuca* (−0.687). The importance of competition was highest on *Festuca* (*R^2^* of 0.719) with high values also on *Arctostaphylos* (0.402) and *Linnaea* (0.477). No species displayed positive density dependence (facilitation).

**Table 3:** Regression coefficients for the relationship between each species mean plant mass and density.

Variable	Model	df	Intercept	Slope	*R^2^*	*P*
*Achillea millefolium* ssp. *borealis*	semilog	53	0.226	−0.119	0.235	**<0.001**
*Arctostaphylos uva-ursi*	power	51	1.965	−0.851	0.402	**<0.001**
*Epilobium angustifolium*	linear	53	2.016	−0.355	0.026	0.247
*Festuca altaica*	power	53	3.892	−0.687	0.719	**<0.001**
*Linnaea borealis*	power	53	3.492	−0.897	0.477	**<0.001**
*Lupinus arcticus*	power	51	−0.762	−0.211	0.161	**0.003**
*Mertensia paniculata*	semilog	53	1.215	−0.263	0.068	*0.056*
*Senecio lugens*	linear	53	0.354	−0.114	0.045	0.124
*Solidago multiradiata*	linear	53	0.424	−0.107	0.063	*0.068*

Significant values (*P*<0.05) are in **bold** and values where *P*<0.10 are in *italics*. The model type is the transformation that best linearized the data. The degrees of freedom (df) are for the model and error combined. These data are plotted in Figure 4.

**Table 4:** Summary of ANCOVAs and ANOVAs on each species' mean plant mass in the CDS in 2002, in response to manipulations of density and fertilizer.

Species	Source	df	SS	*F*-ratio	*P*
*Achillea millefolium* ssp. *borealis*	Density	1	1.231	22.178	**<0.001**
	Fertilizer	2	0.313	2.819	0.071
	Density x Fertilizer	2	0.265	2.389	0.104
	Error	44	2.441		
*Arctostaphylos uva-ursi*	Density	1	53.322	42.204	**<0.001**
	Fertilizer	2	22.371	8.853	**<0.001**
	Density x Fertilizer	2	0.154	0.061	0.941
	Error	44	55.591		
*Epilobium angustifolium*	Fertilizer	2	36.434	10.941	**<0.001**
	Error	51	84.914		
*Festuca altaica*	Density	1	779900	35.754	**<0.001**
	Fertilizer	2	22318	0.512	0.603
	Density x Fertilizer	2	12363	0.283	0.755
	Error	44	959761		
*Linnaea borealis*	Density	1	63.621	57.632	**<0.001**
	Fertilizer	2	9.488	4.297	**0.020**
	Density x Fertilizer	2	3.585	1.624	0.209
	Error	44	48.573		
*Lupinus arcticus*	Density	1	3.261	9.404	**0.004**
	Fertilizer	2	0.462	0.666	0.519
	Density x Fertilizer	2	0.572	0.825	0.449
	Error	44	15.259		
*Mertensia paniculata*	Fertilizer	2	26.201	13.281	**<0.001**
	Error	51	50.305		
*Senecio lugens*	Fertilizer	2	0.620	2.422	0.099
	Error	51	6.532		
*Solidago multiradiata*	Fertilizer	2	0.018	0.101	0.904
	Error	51	4.489		

Significant values (*P*<0.05) are in **bold**. An ANOVA with just fertilizer as an effect was done when the effect of density was not significant (*P*<0.05, Table 3) on the mean plant mass.

**Figure 5:**
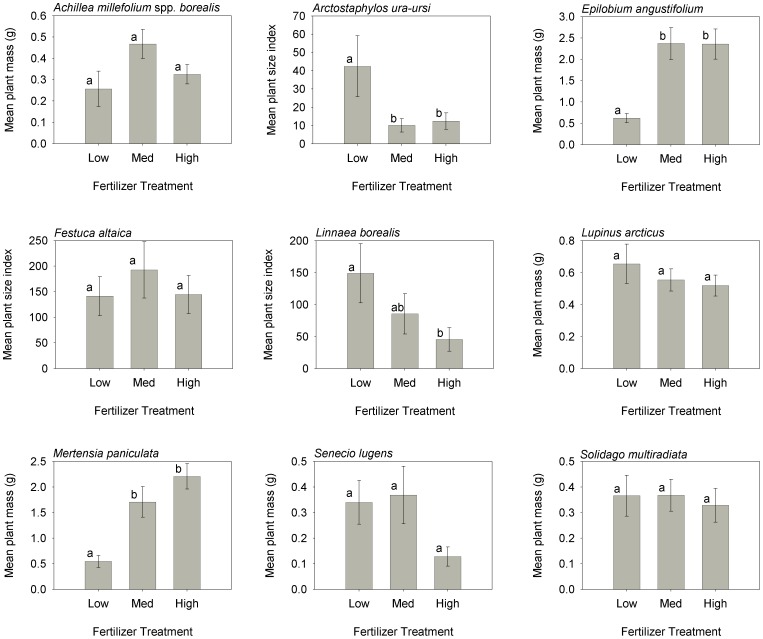
The effect of fertilizer level on the mean plant mass (±1 S.E.) or mean plant size index (±1 S.E.) for each species in the CDS in 2002. Columns sharing the same letter are not statistically different (Tukey's HSD, *P*>0.05).

Species-specific responses to fertilizer addition were more varied than the response to density. The prostrate woody shrubs, *Arctostaphylos* and *Linnaea*, were negatively affected by fertilizer addition while *Epilobium* and *Mertensia* responded favorably to fertilizer addition (Table 4, Fig. 5). The remaining 5 species had no response to fertilizer addition, although *Achillea* and *Senecio* had marginally significant responses (0.05<P<0.10).

A switch between no density dependence to density dependence at low densities was observed for three species, *Achillea*, *Arctostaphylos*, and *Festuca* (Fig. 4). The density that competition began to be important could not be determined for the other species because all densities needed to be included before the regression had a significant slope. Constant yield was reached for three species - for *Achillea*, and *Festuca* at natural field density and for *Lupinus* at 0.5 density.

## Discussion

There are now a number of examples of entire plant communities following the same pattern as observed in single-species experiments that show increasing yield with increasing plant density until an asymptote is reached i.e. the constant final yield [4]–[7] [23] [30]–[31]. Most of these studies were done using annual species in the Negev desert. In common, the Negev studies and the current study show that the initial density had significant effects on final species composition but there was no convergence of species composition or even in functional groups. In contrast, the Negev communities did not converge to a common biomass (after 3 years) whereas our communities did converge to a constant biomass at the natural (1-X) density (after 4 years). Both sets of studies demonstrated that density dependent regulation occurs at the level of the entire community; the current study shows that the intensity and importance of competition increased in each subsequent year but the Negev studies did not make this distinction. Therefore, while both systems showed some degree of convergence of abundance, neither of them showed convergence of species composition.

Knowing whether a plant community is at, or close to, carrying capacity is essential for understanding its behaviour and there is reason to believe that low-density plant communities will behave quite differently, and less predictably, than plant communities close to carrying capacity [2]. At carrying capacity, a community will be using all available resources that act as a limitation on plant community dynamics, increasing predictability [3]. At lower community densities, the plant dynamics are transient and initial conditions and external factors play a crucial role. In the desert, this can be easily understood due to the wide variations of seed-bank density across the landscape, and the variable nature of the species composition of the seed-bank. The spatial variation is influenced by the location and size of adult plants that are dispersing seeds, the nature of the terrain, wind speed and direction when seeds are being scattered, the abundance of seed predators, and other effects. Therefore, the species composition and structure of these annual desert communities of annual plants, although strongly influenced by competition and herbivory, are seemingly indeterminate at the local scale and may be substantially due to chance and unpredictable events. A similar proposal may be made for the boreal perennial community although it must be seen in the framework of a much longer time-scale. Our study area was most recently burned about 80 years before this research. The initial recolonization after the fire would have depended on time of year, location and abundance of species able to disperse into the site, and on the composition of the seed bank. Once established, most of these species rely much more on clonal growth than on seed dispersal and further seedling establishment. After 10 or 20 years, the composition of this community was quite likely still influenced to some extent by the nature of the founder populations. Likewise, after our treatment perturbations, subsequent recolonization may have been strongly influenced by various uncontrolled factors, and even though competition is clearly an important factor structuring this community, chance may also play a role in the structure of the boreal community. Of theoretical significance is that these results indicate that the community response is not simply an additive effect of multiple species responses, but likely also due to history and other interactions that are more complex. When carrying capacity is finally reached, internal processes become more dominant [2]. However, carrying capacity is clearly a function of the plant species composition as well as resource levels. It represents the maximum biomass for a particular assemblage of species in an environment after a period of growth and therefore serves as a baseline for the measurement of disturbance in the community.

Competition began to affect the structure of this experimental community at density levels much lower (x1/8 density) than the natural density and this was apparent in all years of the study. The density at which the community reached constant final yield occurred at the x1 density or natural density in the final two years of the study. In the first two years, constant final yield was not reached likely because insufficient time had elapsed since the densities were manipulated for the mean plant mass to show the effect of competition.

Other than Zamfir and Goldberg [31], this is the only other study to present both species-specific responses and community-level responses using this technique, though many others have reported species diversity changes within a Community Density Series [5] [23] [40]. For competition to significantly affect community structure, and therefore diversity, it must affect species differently [23]. In this experiment, the community as a whole was negatively affected by increasing density with most species showing a decrease in mean plant mass, although two species, *Epilobium* and *Senecio*, were not affected. Similarly, the intensity and importance of competition varied for different species. The effects of competition on the community began with *Festuca* and *Linnaea* at low densities (x1/8), while *Achillea*, *Arctostaphylos* and *Lupinus* did not respond until x1/2. The community reached constant final yield at x1, the natural field density, but only *Achillea* and *Festuca* reached constant final yield (also at x1). No changes in species richness occurred in this experiment, but there were changes in evenness that increased with increasing density. Because evenness expresses how equally abundant species are in a sample, the lowest density plots, or null community without competitive interactions, had some species become much more abundant relative to others, and as density and competition increased, these species were more affected than other species. Evenness was also affected by fertilizer rates with the higher evenness in the fertilized plots. This result is opposite to what has been observed in other research done in this plant community [41] and is contrary to the usual observations [42]–[44]. In addition, competition was neither intense nor relatively important at high productivity for either plant survival or size, suggesting that our results are also not consistent with an increase in the likelihood of competitive exclusion as predicted by Newman, at least not over this range of productivity

Bennett and Cahill's [13] approach focuses on the effects of neighbours on seedling survival and growth. For both seedling survival and growth, relative competitive importance and competitive intensity declined with some measure of productivity; neighbour effects on survival declined with standing crop, while effects on growth declined with gross water supply. These results add to the growing evidence that plant-plant interactions vary among life history components with different life history components contingent upon separate environmental factors [13].

In this boreal understory experiment, the addition of fertilizer had a negative effect on the mean plant mass in the Community Density Series. Another study [45] demonstrated increased productivity in response to fertilizing a low-productivity sand system, but without an effect on diversity. Although the boreal system is generally considered to be nutrient limited, these results are not contradictory to other long-term studies done in this system that have observed both positive and negative effects of increased fertility [34] [41]. Some short-term studies have reported decreases in survival with increased fertilizer [46] while others indicate either no effect of fertilization [47] or positive effects [36]. The species-specific responses to fertilizer addition here correspond well with responses observed by Turkington et al. [41]. Species that decreased with increased fertilizer in both studies include *Arctostaphylos* and *Linnaea*, which are both low-growing prostrate shrubs, while *Epilobium* and *Mertensia*, taller erect herbaceous species, increased in both studies. The biggest difference here is the lack of response of some species that usually increase with added fertilizer such as *Festuca* and *Achillea* [36] [41]. This lack of a positive response, especially for the graminoid, *Festuca*, may in part be due to an unusually high abundance of microtine rodents in 2002 and it is well known that many plants experience increased herbivory when fertilized, especially species growing in low-nutrient environments. There is evidence to suggest that these rodents (voles) may be specifically attracted to the fertilizer added to our experimental plots [48]. Fertilizer level was positively related to the number of over-winter vole nests found in the experimental CDS and unmanipulated plots. There was no evidence to support the idea that fertilizer addition affects the role of competition in structuring this community at either the species or community level, nor was their evidence to support the idea that facilitative interactions may be important in this community.

We have demonstrated that density dependence is important in structuring this boreal understory community utilizing the community density series. This CDS approach allows us to quantify both the intensity and importance of plant competition at the community and species levels and to determine whether the importance of these biotic interactions depend on abiotic factors. While fertilizer addition did have minor effects on the community, it did not change the intensity of competition. The results presented here clearly show that species-specific responses to biotic interactions are not necessarily the same as community level ones and if we are to understand what occurs at the community level, it is necessary to use appropriate methodological approaches such as the CDS.

## Supporting Information

Table S1 Initial abundance of species. The abundance of all species found in the 63 1 m^2^ Community Density Series plots during the initial survey in 1999. Frequency is the percent occurrence in the 63 plots. Percent cover was estimated using a point frame with 100 pin drops per m^2^. Density was assessed by counting all individuals in the 1 m^2^ plot. The densities of *Arctostaphylos uva-ursi*, *Festuca altaica* and *Linnaea borealis* were not estimated *(n/a)* due to the impossibility of identifying distinct individuals.
(DOC)

Table S2 Equations used to estimate plant biomass for all species. The equations used to estimate biomass for the years 1999 through 2001 for all species growing in the Community Density Series plots and control plots. These equations were the best fitting curves between biomass and various surrogates for biomass and were based on destructive sampling done in 1999. In the equations: C  =  cover, H  =  height, L  =  length of the longest leaf, N  =  number of leaves, and W  =  width of longest leaf. All equation components are measured in mm and all estimated masses are in grams.
(DOC)

Table S3 Density and biomass estimates for all species in all plots for all years.
(XLS)
