# Supplementary material for: Species-Specific Responses to Community Density in an Unproductive Perennial Plant Community
Source: PLoS One. 2014 Jul 22;9(7):e102430. doi: 10.1371/journal.pone.0102430 (PMC4106790; doi:10.1371/journal.pone.0102430)
Supplement: Table S1 — Initial abundance of species. The abundance of all species found in the 63 1 m2 Community Density Series plots during the initial survey in 1999. Frequency is the percent occurrence in the 63 plots. Percent cover was estimated using a point frame with 100 pin drops per m2. Density was assessed by counting all individuals in the 1 m2 plot. The densities of Arctostaphylos uva-ursi, Festuca altaica and Linnaea borealis were not estimated (n/a) due to the impossibility of identifying distinct individuals. (DOC) [file pone.0102430.s001.doc]

**Table S1. Initial abundance of species**

The abundance of all species found in the 63 1 m^2^ Community Density Series plots during the initial survey in 1999. Frequency is the percent occurrence in the 63 plots. Percent cover was estimated using a point frame with 100 pin drops per m^2^. Density was assessed by counting all individuals in the 1 m^2^ plot. The densities of *Arctostaphylos uva-ursi*, *Festuca* *altaica* and *Linnaea* *borealis* were not estimated *(n/a)* due to the impossibility of identifying distinct individuals.

| Species | Frequency (%) | Mean percent cover ± S.E. | | Mean density (no./m^2^) ± S.E. | |
| --- | --- | --- | --- | --- | --- |
| *Achillea millefolium* ssp. *borealis* | 84.13 | 1.58 | ± 0.26 | 21.97 | ± 2.83 |
| *Anenome parviflora* | 11.11 | 0.79 | ± 0.31 | 8.14 | ± 3.15 |
| *Antennaria* spp. | 31.75 | 0.18 | ± 0.04 | 1.11 | ± 0.27 |
| *Arabis* spp. | 3.17 | 0.02 | ± 0.01 | 0.06 | ± 0.05 |
| *Arctostaphylos rubra* | 7.94 | 0.67 | ± 0.57 | 5.83 | ± 4.73 |
| *Arctostaphylos uva-ursi* | 58.73 | 6.26 | ± 1.29 | *n/a* |  |
| *Artemisia norvegica* | 6.35 | 0.06 | ± 0.04 | 0.90 | ± 0.68 |
| *Aster* spp. | 1.59 | 0.01 | ± 0.01 | 0.03 | ± 0.03 |
| *Betula* spp. | 11.11 | 0.10 | ± 0.04 | 0.24 | ± 0.10 |
| *Calamagrostis* spp. | 25.40 | 0.15 | ± 0.04 | 2.43 | ± 0.82 |
| *Carex* spp. | 23.81 | 0.25 | ± 0.07 | 3.87 | ± 1.20 |
| *Cornus canadensis* | 20.63 | 0.28 | ± 0.10 | 3.05 | ± 1.11 |
| *Delphinium glaucum* | 14.29 | 0.10 | ± 0.04 | 0.27 | ± 0.10 |
| *Draba* spp. | 7.94 | 0.04 | ± 0.02 | 0.57 | ± 0.30 |
| *Epilobium angustifolium* | 53.97 | 1.17 | ± 0.25 | 3.54 | ± 0.65 |
| *Festuca altaica* | 100.00 | 14.21 | ± 1.25 | *n/a* |  |
| *Gentiana* spp. | 39.68 | 0.25 | ± 0.05 | 7.64 | ± 2.64 |
| *Linnaea borealis* | 90.48 | 25.33 | ± 2.68 | *n/a* |  |
| *Lupinus arcticus* | 85.71 | 2.68 | ± 0.42 | 27.38 | ± 4.59 |
| *Mertensia paniculata* | 57.14 | 3.29 | ± 0.81 | 12.59 | ± 2.44 |
| *Moneses uniflora* | 20.63 | 0.14 | ± 0.04 | 1.67 | ± 0.59 |
| *Orthilia secunda* | 11.11 | 0.14 | ± 0.07 | 1.14 | ± 0.49 |
| *Pedicularis* spp. | 1.59 | 0.01 | ± 0.01 | 0.02 | ± 0.02 |
| *Picea glauca* | 12.70 | 0.16 | ± 0.07 | 0.16 | ± 0.06 |
| *Polemonium* spp. | 1.59 | 0.03 | ± 0.03 | 0.03 | ± 0.03 |
| *Pyrola* spp. | 1.59 | 0.02 | ± 0.02 | 0.02 | ± 0.02 |
| *Salix* spp. | 3.17 | 0.02 | ± 0.01 | 0.05 | ± 0.04 |
| *Senecio lugens* | 80.95 | 1.36 | ± 0.21 | 8.52 | ± 1.22 |
| *Shepherdia canadensis* | 9.52 | 0.07 | ± 0.04 | 0.21 | ± 0.11 |
| *Solidago multiradiata* | 90.48 | 1.20 | ± 0.15 | 11.97 | ± 1.36 |
| *Stellaria longipes* | 4.76 | 0.02 | ± 0.01 | 0.49 | ± 0.34 |
| *Trisetum spicatum* | 7.94 | 0.06 | ± 0.03 | 0.79 | ± 0.42 |
