# Supplementary material for: Species-Specific Responses to Community Density in an Unproductive Perennial Plant Community
Source: PLoS One. 2014 Jul 22;9(7):e102430. doi: 10.1371/journal.pone.0102430 (PMC4106790; doi:10.1371/journal.pone.0102430)
Supplement: Table S2 — Equations used to estimate plant biomass for all species. The equations used to estimate biomass for the years 1999 through 2001 for all species growing in the Community Density Series plots and control plots. These equations were the best fitting curves between biomass and various surrogates for biomass and were based on destructive sampling done in 1999. In the equations: C = cover, H = height, L = length of the longest leaf, N = number of leaves, and W = width of longest leaf. All equation components are measured in mm and all estimated masses are in grams. (DOC) [file pone.0102430.s002.doc]

Table S2. Equations used to estimate plant biomass for all species.

The equations used to estimate biomass for the years 1999 through 2001 for all species growing in the Community Density Series plots and control plots. These equations were the best fitting curves between biomass and various surrogates for biomass and were based on destructive sampling done in 1999. In the equations: C = cover, H = height, L = length of the longest leaf, N = number of leaves, and W = width of longest leaf. All equation components are measured in mm and all estimated masses are in grams.

| Species | Plant form | Biomass equation | n | R^2^ | *P* |
| --- | --- | --- | --- | --- | --- |
| *Achillea millefolium* ssp. *borealis* | non-flowering | L x 0.0005 | 34 | 0.816 | <0.0001 |
|  | flowering | H x 0.001 | 10 | 0.701 | 0.0025 |
| *Arctostaphylos uva-ursi* | *n/a* | C x 3.234 | 17 | 0.637 | <0.0001 |
| *Epilobium angustifolium* | non-flowering | H x 0.0021 | 17 | 0.672 | <0.0001 |
|  | flowering | H x 0.0032 | 6 | 0.974 | 0.0003 |
| *Festuca altaica* | *n/a* | C x 1.099 | 17 | 0.878 | <0.0001 |
| *Linnaea borealis* | *n/a* | C x 2.013 | 16 | 0.867 | <0.0001 |
| *Lupinus arcticus* | non-flowering | W x 0.0045 | 19 | 0.618 | <0.0001 |
|  | flowering | W x 0.0065 + H x 0.0005 | 11 | 0.800 | <0.0001 |
| *Mertensia paniculata* | non-flowering | L x 0.0011 | 21 | 0.814 | <0.0001 |
|  | flowering | H x 0.0012 | 14 | 0.808 | <0.0001 |
| *Senecio lugens* | non-flowering | N x 0.016 + L x 0.0006 | 21 | 0.870 | <0.0001 |
|  | flowering | L x 0.0009 + H x 0.0019 | 9 | 0.745 | 0.0165 |
| *Solidago multiradiata* | non-flowering | L x 0.0011 | 20 | 0.870 | <0.0001 |
|  | flowering | L x 0.021 + H x 0.001 | 10 | 0.658 | 0.0234 |
